# Emergency department-based medication review on outpatient health services utilization: interrupted time series

**DOI:** 10.1186/s12913-020-05108-6

**Published:** 2020-03-26

**Authors:** Sophie A. Kitchen, Kimberlyn McGrail, Maeve E. Wickham, Michael R. Law, Corinne M. Hohl

**Affiliations:** 1 School of Population and Public Health, 2206 East Mall, Vancouver, BC V6T 1Z9 Canada; 2 Centre for Health Services and Policy Research, 201-2206 East Mall, Vancouver, BC Canada; 3 Centre for Clinical Epidemiology & Evaluation, Vancouver Coastal Health Research Institute, 900 West 10th Ave, Vancouver, BC V5Z 1M9 Canada; 4 Department of Emergency Medicine, University of British Columbia, 855 West 12th Avenue, Vancouver, BC V5Z 1M9 Canada

**Keywords:** Medication review, Adverse drug events, Pharmacy, Health services

## Abstract

**Background:**

One in nine emergency department (ED) visits in Canada are caused by adverse drug events, the unintended and harmful effects of medication use. Medication reviews by clinical pharmacists are interventions designed to optimize medications and address adverse drug events to impact patient outcomes. However, the effect of medication reviews on long-term outpatient health services utilization is not well understood. This research studied the effect of medication review performed by clinical pharmacists on long-term outpatient health services utilization.

**Methods:**

Data included information from 10,783 patients who were part of a prospective, multi-centre quality improvement evaluation from 2011 to 2013. Outpatient health services utilization was defined as total ED visits and physician contacts, aggregated to four physician specialty groups: general and family practitioners (GP); medical specialists; surgical specialists; and imaging and laboratory specialists. During triage, patients deemed high-risk based on their medical history, were systematically allocated to receive either a medication review (*n* = 6403) or the standard of care (*n* = 4380). Medication review involved a critical examination of a patient’s medications to identify and resolve medication-related problems and communicate these results to community care providers. Interrupted time series analysis compared the effect of the intervention on health services utilization relative to the standard of care controlling for pre-intervention differences in utilization.

**Results:**

ED-based pharmacist-led medication review did not result in a significant level or trend change in the primary outcome of total outpatient health services utilization. There were also no differences in the secondary outcomes of primary care physician visits or ED visits relative to the standard of care in the 12 months following the intervention. Our findings were consistent when stratified by age, hospital site, and whether patients were discharged on their index visit.

**Conclusion:**

This was the first study to measure long-term trends of physician visits following an ED-based medication review. The lack of differences in level and trend of GP and ED visits suggest that pharmacist recommendations may not have been adequately communicated to community-based providers, and/or recommendations may not have affected health care delivery. Future studies should evaluate physician acceptance of pharmacist recommendations and should encourage patient follow-up to community providers.

## Background

One in nine emergency department (ED) visits are caused by an adverse drug event (ADE), the unintended and harmful effects of medication use [1, 2]. ADEs are associated with greater inpatient and outpatient health services utilization and costs, and are between the fourth and sixth leading cause of death in North America [3–6]. Unfortunately, it is often difficult to detect and address ADEs in clinical encounters. Up to 50% of ADEs are misdiagnosed by physicians in EDs and on hospital wards, leading to treatment delays and lack of withdrawal or replacement of culprit medications [7–9]. Furthermore, a recent study estimated that up to 55% of older adults admitted to hospital with ADEs are re-exposed to the potentially causative medications within 6 months of discharge [10]. Finding effective interventions to improve early detection and treatment, and effective communication of inappropriate medication therapy and ADEs, has the potential to reduce unnecessary downstream health services utilization and avoid preventable patient harm.

Medication review is one intervention proposed to maximize the benefit of medications, while limiting their potential for harm. Medication review is an in-person, structured, critical examination of a patient’s medications performed by a qualified healthcare provider, typically a pharmacist [11]. Medication review goes a step beyond the standard of care, medication reconciliation, to not only carefully assess and document medications, but also to have health care providers think critically about how to optimize those medications to minimize medication-related problems and then communicate their findings with the patient, the patient’s family or caregiver, or community-based care providers [12]. While medication reviews have been tested and evaluated in primary care and hospital settings, few studies have evaluated the effect of pharmacist-led medication review among patients in the ED-setting [13].

A previous evaluation of the intervention under study measured the effect of pharmacist-led medication review among high-risk ED patients on the number of days patients spent in hospital [14]. Post intervention, the patients in the medication review group spent a median of 0.48 days (95% CI: 0.00 to − 0.96, *p* = 0.058) less in hospital within 30 days of the index ED visit compared to patients in the control group who received medication reconciliation, representing a 8% reduction in the length of hospital stay. While secondary outcomes included ED revisits within 7 days, unplanned hospital readmissions, and all-cause mortality, these analyses were limited in follow-up time, and did not account for a potential effect of medication review on outpatient physician visits. Therefore, the present study evaluated the effect of ED-based pharmacist-led medication review in patients at high risk of presenting to the ED with an ADE on trends of outpatient health services utilization compared to standard care, which was medication reconciliation.

## Methods

### Design and setting

This was a population-based evaluation of a continuous quality improvement project using administrative health data. During the quality improvement project, the EDs of one tertiary care referral centre (Vancouver General, VGH), and two urban community hospitals (Lions Gate, LGH; and Richmond General, RH) implemented an evidence-based screening tool, which categorized patients into high and low-risk for adverse drug events based on their medication use, preexisting medical problems, and age [15, 16]. Pharmacists subsequently reviewed the medications of high-risk patients in the ED [17].

Following a 6- to 8-week pilot phase, a 12-month evaluation period began at two sites, and a 3-month evaluation at one site (due to staffing constraints) between November 2011 and January 2013. Implementing the medication review required additional resources beyond the standard-of-care. Funding to implement the program allowed for additional clinical pharmacists (hereafter referred to as “pharmacists”), beyond the regular ED pharmacist, to be hired to conduct medication reviews. As implementation of the program would augment the existing standard of care, it was deemed ethical to create a control group of patients who received standard care.

The University of British Columbia Clinical Research Ethics Board reviewed the study protocol and deemed it to be the evaluation of a quality improvement initiative and waived the need for informed consent.

### Participants

We included all patients who were categorized as at high-risk of presenting with an ADE according to a validated decision rule [15, 16], who were 19 years of age or older, and presented to a participating ED when a clinical pharmacist was on shift [18]. We excluded patients with a Canadian Triage Acuity Score (CTAS) of 1 (i.e.*,* resuscitation; requiring immediate physician assessment), multisystem trauma (e.g.*,* penetrating trauma), scheduled visits, sexual assaults, postsurgical or pregnancy-related complications, social problems, and duplicate visits (e.g., repeat high-risk visit after a first high-risk visit), as well as those who died on arrival, left against medical advice, or lived out-of-province. We excluded any months of individuals’ data following their death during the 12-month follow-up.

There were insufficient funds to schedule pharmacists around the clock. Therefore, pharmacists delivered the intervention on the days of the week and during times of highest volume of high-risk patients at each site, based on administrative data collected during the pilot period. Pharmacist coverage varied from a minimum of eight hours per day on weekends and holidays at all sites, to 12 h per day (two sites), and 16 h per day (one site) on weekdays. We provided double and triple coverage of pharmacists during the busiest hours and days of the week, and did not cover night times at any sites, as the lowest number of high-risk patients presented at night.

### Study enrolment and group allocation

We designed a patient enrolment and group allocation algorithm that enabled pharmacists to complete the maximum possible number of medication reviews, while creating two comparable groups of patients for evaluation. We described this protocol in detail in our published protocol [17]. Our funders (The BC Ministry of Health and Vancouver Coastal Health Authority) specified that we were not allowed to randomize patients, and that we must maximize the number of patients receiving the intervention. Given the fixed number of available pharmacists to deliver the intervention, three pre-identified factors created a random availability of pharmacists at any given point in time: *i)* a variable influx of high-risk patients, *ii)* a constant pressure to discharge lower-acuity patients, and *iii)* a variable amount of time required to complete each medication review [17]. At the beginning of each shift, pharmacists sorted the ED census by the time of patient arrival. Pharmacists would then determine the highest possible ratio of patients that could be allocated to the intervention relative to control, based on the number of high-risk patients waiting to be seen and the available pharmacist resources. They determined that ratio at the beginning of their shift (e.g. 1:1, 2:1, 3:1), and applied the ratio to the sorted list of patients. They always allocated the first eligible patient to intervention, and subsequent eligible patients to intervention or control based on the sequence of their arrival time, and the predetermined ratio of intervention to control patients.

If a pharmacist missed an eligible patient during their data collection shift because the patient had already been discharged, the pharmacist re-sorted the ED census. The pharmacist did this by sorting the ED census by the time of patient arrival. After sorting the census during their shift, the pharmacist enrolled the first eligible patient presenting within the past hour of their shift, and allocated them to medication review. If a pharmacist needed to sort the census during their shift, they were asked to adjust the ratio of medication review to control patients downward to minimize any future missed eligible patients.

### Intervention and control

#### Intervention

In the intervention group, pharmacists completed a medication review. This was a structured, in-person critical examination of a patient’s medications which included obtaining a best-possible medication history using multiple sources, reaching agreement with the patient about treatment goals, optimizing medications, and identifying and addressing any medication-related problems and ADEs the patient presented with [11]. Pharmacists focused their reviews on documenting and ensuring appropriate treatment of ADEs, and communicating medication-related problem and ADEs to admitting care providers and family physicians using written notes in patients’ hospital charts, phone calls and by faxing recommendations to family physician offices [12]. Pharmacists documented the ADE diagnosis in the hospitals’ electronic patient information system. Pharmacists conducting the reviews were residency-trained with a minimum of 2 years’ working experience in an acute care hospital.

#### Control

All participating hospitals had implemented medication reconciliation as standard care at the time the quality improvement project started. Medication reconciliation is the process of creating a complete and accurate list of a patient’s current medications, including the name, dosage, frequency, and route of administration using multiple sources including the patient, family, community pharmacists to avoid medication errors at transitions in care [19]. Physicians or nurses completed medication reconciliation in control patients, and consulted the regular ED clinical pharmacist if needed, for any questions they had or were unable to resolve regarding the patient’s medication management.

### Data sources

We completed longitudinal analyses using de-identified administrative health data. We used the following databases: PharmaNet, which captures all prescription drug dispensations in British Columbia, Medical Services Plan, which contains all fee-for-service physician encounters in British Columbia, Discharge Abstract Database, which contains national data on hospital admissions, discharges, transfers, and deaths of patients from acute care hospitals and Vital Statistics, which contains data on deaths in the province [20–23]. These data sources provide comprehensive health services information covering approximately 95% of the population, excluding federally insured populations (e.g. First Nations, police, and veterans).

### Outcome variables

The primary outcome was the total number of physician visits per 1000 patients per month for 12 months following the index visit, aggregated to four physician specialty groups: general and family practitioners; medical specialists; surgical specialists; and imaging and laboratory specialists. Secondary outcomes included general practitioner visits per 1000 patients per month, and ED visits per 1000 patients per month. We recorded all outcomes per person-month for each patient from 12 months before to 12 months after the intervention, and per person-week as 52 weeks before and after the intervention. We performed a priori planned subgroup analyses to examine patient-level factors affecting the likelihood of these outcomes; specifically, analyses for < 80 years of age, > 80 years of age, whether the patient was admitted to the hospital on the index visit, and by hospital site. We also completed sensitivity analyses, as we observed substantially increased health services visits at time point 0, the first month following the medication review. Although the increase in visits was expected, including these points in the model distorted the trends of visits. Therefore, we conducted post hoc sensitivity analyses with these points excluded from the models, and with an indicator term included to adjust for the outliers at time points − 1 and 0. Both approaches similarly adjusted for the expected outliers, and therefore, we excluded time points − 1 and 0 from the model of health services outcomes. We also matched the intervention and control groups using propensity scores based on age, gender, CTAS score, number of medications 6 months prior to intervention, arrival mode, and arrival time to test for residual confounding.

### Statistical analysis

We completed unadjusted bivariate statistics using the two-sample t-test or the Wilcoxon rank-sum test or Chi-square test if the normality assumption was not met. We used interrupted time series analyses to assess the effect of medication review on the primary outcomes at 24 distinct time points, 12 months before and after the intervention in study-time [24]. Multiple visits to the same practitioner-type on the same day were considered as one unique visit, with the exception of ED visits in which each visit per day was considered as a unique visit. We prepared the data with SAS, version 9.4 and completed all statistical analyses with R statistical software package, version 3.5.2.

## Results

### Descriptive and bivariate statistics

**Fig. 1:**
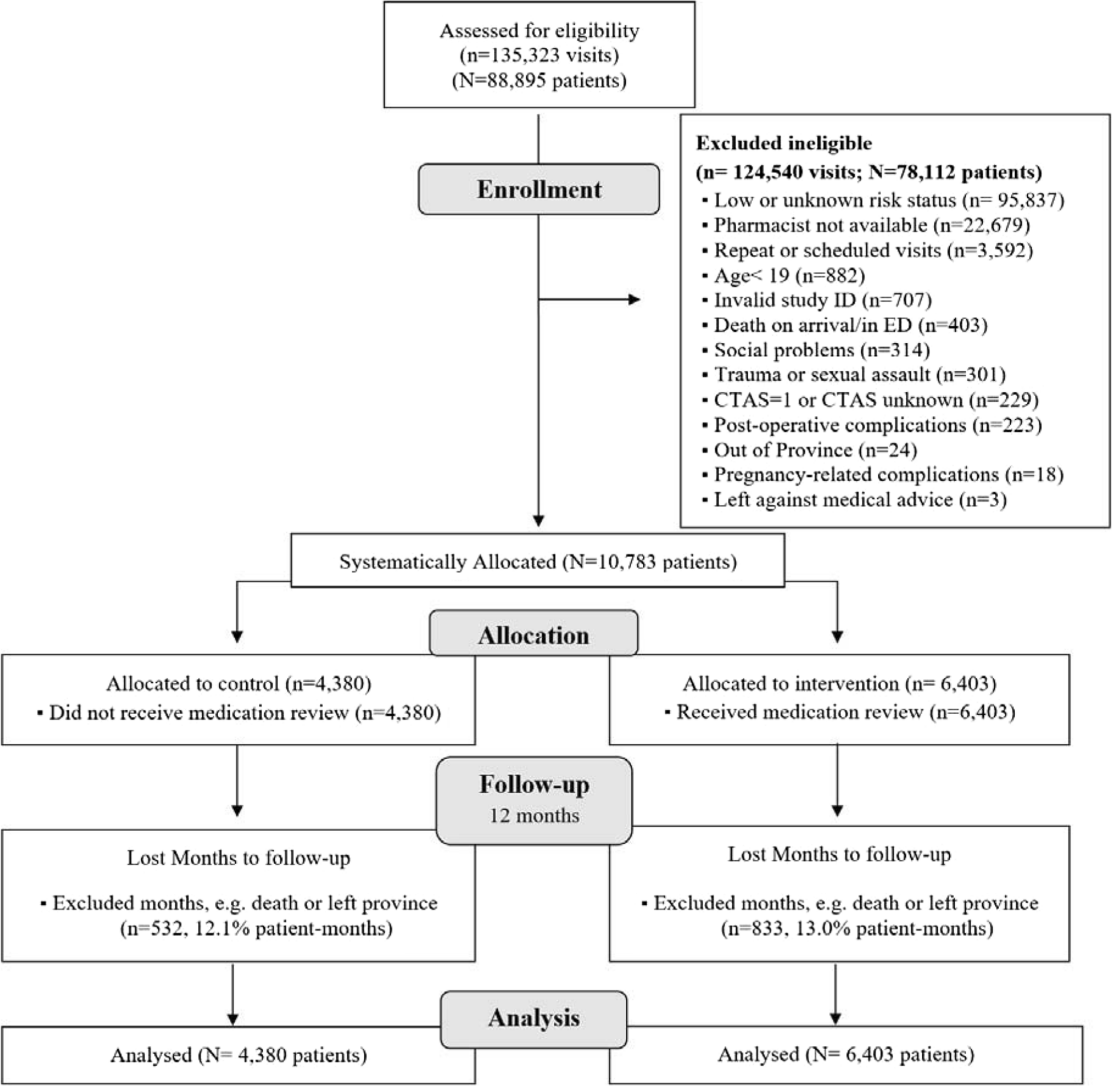
Flow diagram of patients allocated to receive a medication review or control

Between December 2011 and April 2012, 88,895 patients made 135,323 ED visits at the three participating hospital sites. We excluded 124,516 patient visits (Fig. 1), most because they were categorized as being at “low-risk” risk for an ADE-related presentation (93,453, 75%). Of the remaining 10,783 eligible patient visits, 6403 were systematically allocated to intervention, and 4380 were allocated to control. During the 12-month follow-up time, 13.0% of patient-months in the intervention and 12.1% of patient-months in the control were excluded from the analysis due to death or leaving the province.

When comparing baseline characteristics between groups, the intervention group contained a higher proportion of individuals enrolled at VGH (74.7%), which is the highest acuity hospital among the three participating sites, relative to control (60.5%; *p* < 0.001, Table 1). The median age of those in the intervention was 71 (IQR: 31) relative to 69 in the control (IQR: 33; *p* = 0.006), and the intervention had an average of 8.4 active medications (SD = 5.8) compared to 8.1 in the control (SD = 5.8; *p* = 0.02). There were no significant differences between groups with regards to sex, admission, or CTAS score on index visit.

**Table 1:** Descriptive statistics of the overall study sample, and by study group. Significant differences between groups were measured using a two sample t-test or the appropriate nonparametric test (Wilcoxon rank-sum test or Chi-square test) if the normality assumption was not met

	Overall Study Sample	Treatment Allocation	
	10,783 (100%)	Yes (%)	No (%)
**Medication Review**			
Yes	6403 (59.4)	59.4	40.6
**Hospital**			
VGH	7434 (68.9)	74.7***	60.5***
LGH	2676 (24.8)	17.6***	26.3***
RH	673 (6.2)	7.7***	4.1***
**Sex**			
Female	6031 (55.9)	56.5	55.2
Male	4752 (44.1)	43.5	44.8
**Age**			
Median (IQR)	70 (32)	71 (31)**	69 (33)**
19–44 years	1904 (17.7)	16.9	18.8
45–64 years	2703 (25.0)	24.6	25.8
65–79 years	2326 (21.6)	21.8	21.1
80–105 years	3850 (35.7)	36.7	34.3
**CTAS**			
2 (Emergency)	2553 (23.7)	23.2	24.3
3 (Urgent)	6241 (57.9)	58.3	57.2
4 (Semi-Urgent)	1896 (17.6)	17.6	17.5
5 (Non-Urgent)	93(0.9)	0.8	0.9
**Number of Active Medications**^**a**^			
Mean (SD)	8.3 (5.8)	8.4 (5.8)*	8.1 (5.8)*
**Discharged on index visit**			
Discharged	6544 (60.7)	60.3	61.3
Admitted	4239 (39.3)	39.7	38.7

^**a**^Within 6 months of index visit, *indicates *p* < 0.05, ** *p* < 0.01, and ***p < 0.001, by a two sample t-test

### Outpatient health services utilization

We used interrupted time series to determine the effect of the intervention compared to control while adjusting for pre-intervention health services utilization. At baseline (12 months before the intervention), there was no difference in total physician visits between intervention (2565 visits per 1000 patients; 95% CI: 2095.9, 3023.4; *p* = 0.64) and control (2454 visits per 1000 patients, 95% CI: 2129.9, 2778.6); Table 2). There was a trend of increasing pre-intervention physician visits per month for both intervention (57.1 visits per 1000 patients, 95% CI: − 5.6, 119.8) and control (61.5 visits per 1000 patients, 95% CI: 17.2, 105.9, which was not different between groups (*p* = 0.64). Similarly, there was no difference in pre-intervention levels of visits between groups (*p* = 0.89). At 12 months, there was no change in the level or trend of total physician visits per 1000 patients between groups. Sensitivity analyses, including propensity score matching, did not reveal any differences across groups and were consistent with the finding of no differences between groups.

**Table 2:** Health Services Utilization Model Results

	Total Physician Visits (Visits per 1000 patients)	GP Visits (Visits per 1000 patients)	ED Visits (Visits per 1000 patients)
Visits pre-intervention (control)	2454.3 (2129.92778.6; *p* < 0.001)	1056.4 (913,1199.9; *p* < 0.001)	107.1 (83.4130.8; *p* < 0.001)
Trend pre-intervention (control)	61.5 (17.2105.9; *p* = 0.01)	21.1 (1.7,40.5; *p* = 0.04)	3.6 (0.3,6.9; *p* = 0.037)
Baseline difference in visits between intervention and control	110.3 (− 348.4569.1; *p* = 0.64)	26.2 (− 176.6229.1; *p* = 0.8)	−5.3 (−38.8,28.3; *p* = 0.76)
Differential trend between intervention and control (0–12 months pre-intervention)	− 4.4 (− 67.1,58.3; *p* = 0.89)	−1.6 (− 29.1,25.8; *p* = 0.908)	2.1 (−2.6,6.7; *p* = 0.392)
Change in visits from pre to post-intervention (control)	1488.2 (1181.31795; *p* < 0.001)	612.2 (483.5740.9; *p* < 0.001)	47.9 (23.7,72.1; *p* = 0.004)
Trend change from pre to post-intervention (control)	− 384.5 (− 455.5,-313.6; *p* < 0.001)	− 152.3 (− 183.6,-121; *p* < 0.001)	− 16.6 (− 21.7,-11.4; *p* < 0.001)
**Change in visits post-intervention between intervention and control**	**163.6** **(− 270.4597.5;*p*** **= 0.46)**	**113.4** **(− 68.6295.4;*p*** **= 0.23)**	**−7.6** **(− 41.8,26.6;*p*** **= 0.67)**
**Trend change post-intervention between intervention and control**	**7.1** **(− 93.3107.4;*p*** **= 0.89)**	**−2.2** **(− 46.5,42.1;*p*** **= 0.92)**	**−2.1** **(− 9.4,5.2;*p*** **= 0.57)**

Although patients in both intervention and control groups experienced a sharp increase in GP visits following their index ED visit, there were no significant differences in the level or trend of GP visits following the intervention, including when we stratified the results by age. GP visits accounted for 69.3% of the level increase in total physician visits among the entire population.

**Fig. 2:**
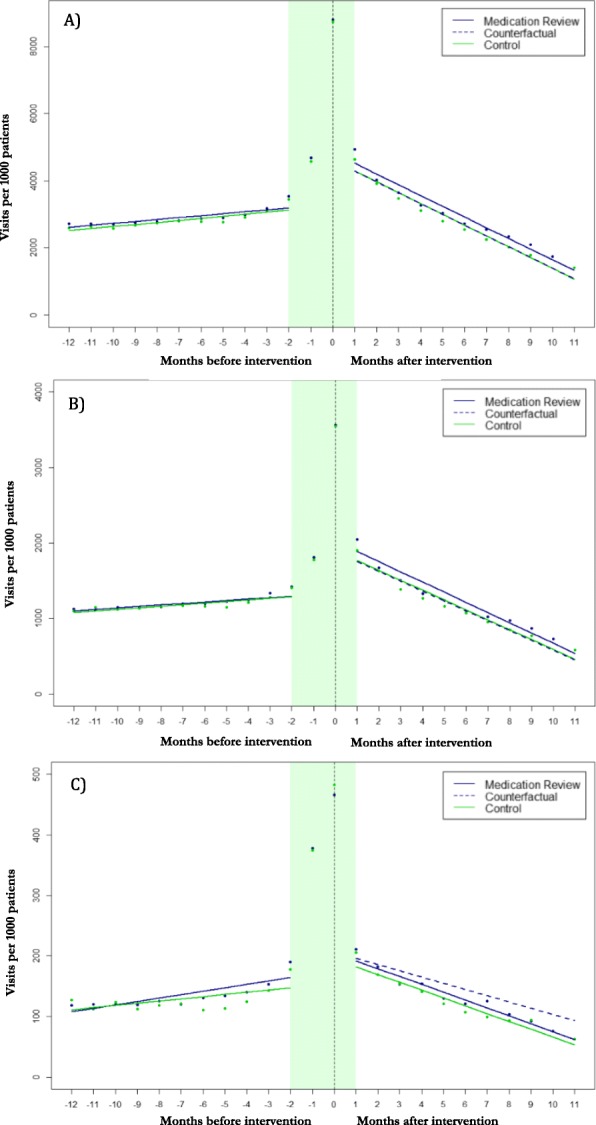
Number of (**a**) total outpatient, (**b**) ED, (**c**) GP visits per 1000 patients allocated to medication review or control

There was no difference in the level or trend of the number of ED visits per 1000 patients between groups in 12 months of follow-up. While we observed a decrease of 30 ED visits per 1000 patients (95% CI: − 61.2, 2.5) in the intervention group compared to control among patients over 80 in the month following the index visit, but this difference was not statistically significant (*p* = 0.08; Fig. 2, Table 2). This effect was attenuated when we stratified the analysis by ED disposition.

## Discussion

Our objective was to evaluate the effect of an ED-based pharmacist-led medication review intervention on health services utilization using an interrupted time-series design. Our results indicate that the intervention did not modify long-term trends of total physician, family physician or ED visits, even when we stratified our results by age, discharge status, or by hospital site.

The lack of observed differences in GP visits between the intervention and control groups is consistent with prior literature in the field, with three prior hospital-based studies indicating no effect, and one indicating a modest increase in GP and urgent care visits following the intervention [25–28]. While Okere et al. observed an increase in GP visits among patients who received ED-based medication review, their study enrolled a much younger patient population than others [25–28]. The patients included in the present study were older, with a median age of 70 years. As older patients are more likely to return to residential care or be admitted to hospital after an ED visits, they may be less likely to follow up on the result of a medication review with a GP in the community [29, 30]. As a result, outpatient health services utilization outcomes may be less discerning in this older patient population. In addition, while ADEs are common in older adults with high levels of morbidity, their subsequent health services utilization may be driven by factors other than more rapid resolution of medication-related problems or preventative interventions, such as their frailty, lack of social supports and loss of independence [31].

This may lead to smaller discernible differences between groups in a controlled study attempting to measure the effect of medication review in older ADE patients on health services utilization.

We observed no differences in level or trend of ED revisits per 1000 patients following medication review. In light of the primary study, which indicated a clinically important reduction in the number of hospital days in the intervention group compared to control, our finding of no difference in repeat ED visits is reassuring, and indicates that patients in the intervention group were not inappropriately discharged earlier [14].

### Strengths and limitations

Our study was a non-randomized, non-blinded controlled clinical trial. In order to mitigate the inherent risk of bias in non-randomized designs we used an interrupted time series approach, allowing us to confirm that there were no pre-intervention differences in the level or trend of any of the health outcomes we measured. This indicates that the systematic allocation algorithm was successful in creating comparable groups of patient. We enrolled a large sample size of patients under real-world circumstances and completed our evaluation using exclusively administrative health data. This provided us the opportunity to evaluate a medication review as implemented outside of the highly controlled environment of randomized trials. In addition, we compared medication review to medication reconciliation with as needed pharmacist consultation, improving the external validity of our findings relative to other studies, which used no intervention as a control. It is unlikely in acute care settings today, that a control group of patients would not receive any medication management interventions, as medication reconciliation is considered standard care in acute care [12]. Therefore, our results are likely generalizable to other provinces in Canada, and other countries with similar healthcare systems.

Future studies evaluating clinical pharmacy interventions when randomized trials are not ethically feasible or too costly, may consider using a similar design, particularly when health outcome trends over time are relevant. Unlike previous studies, the measurement of trends using this approach controlled for outliers, which may not have been representative of a true change in the outcome [32].

This study is not without limitations. Several studies have previously measured the appropriateness of pharmacist recommendations, and have found that pharmacists’ recommendations are generally appropriate and clinically relevant [33, 34]. We did not implement the use of any implicit or explicit tools to identify inappropriate medications (e.g. MAI or STOPP/ START checklist), as the focus of our ED-based intervention was the identification of ADEs, and many ADEs occur in appropriately prescribed and administered medications (e.g., hypoglycemia in a patient with diabetes who was on insulin). As few tools have been developed and validated to standardize ED-based medication review in patients presenting with ADEs, we were unable to standardize the intervention, aside from emphasizing the clinical focus on identifying and treating ADEs bringing patients into hospital. Research has shown that explicit tools may not be effective at identifying all types of ADEs in ED patients, and could restrict the medication review and limit its potential impact [35–38]. Including both implicit and explicit review criteria may capture the benefits of highly standardized approaches while ensuring that clinicians are empowered to make clinical decisions and provide individualized care [39].

Recently, an international core outcome set released seven recommended outcomes to measure medication review [40]. These outcomes suggested that researchers examine adverse events using drug-related hospitalizations. While drug-related ED visits and hospitalizations would have been ideal outcomes for our study drug-related causality is consistently underreported in administrative data, and therefore not useful [41].

This study was unable to assess whether recommendations made by clinical pharmacists in the ED were successfully communicated to and adopted by GPs. If recommendations were not adopted, our finding of no difference across all measures of subsequent outpatient health services utilization may indicate ineffective communication across health sectors. Communication and acceptance of pharmacist recommendations have been identified as a threat to the success of medication review interventions [25, 39, 42]. A recent study by Santolaya-Perrín et al. trialed four different communication techniques on the uptake of physician acceptance of pharmacists’ recommendations [39]. The technique most similar to the approach we used in our study led to an acceptance of only 27% of recommendations. In contrast, a site that used electronic clinical record systems observed an acceptance rate of 52%. Recently, a separate survey found that 96.7% of GPs stated electronic communication of medication recommendations as their preferred method of receiving prescribing information [43].

The effectiveness of technological solutions for communicating between care providers may continue to improve with growing use and could provide a more cost-effective pathway for information exchange than face-to-face interactions [44]. Future research is needed to develop and evaluate communication strategies to ensure the uptake of pharmacist recommendations, while evaluating their impact on health outcomes.

## Conclusions

As currently designed, medication review compared to a medication reconciliation intervention in which healthcare providers could consult ED pharmacists as needed did not result in long-term changes to outpatient health services utilization. This may be a reflection of the setting in which the medication review took place: in the ED, the goals of care generally focus on the acute problem bringing a patient to hospital, as opposed to preventative longer-term medication management decisions. Additionally, once admitted to hospital, both groups of patients had access to ward-based pharmacists, and thus patients in the control group may have received medication review during their admission. Thus, among admitted patients our finding of no differences between groups in outpatient health services utilization is not surprising, and this dilutes the signal-to-noise ratio in the full population. Another possible explanation for our findings is that community-based care providers may not have adopted ED pharmacist recommendations. Among patients discharged from the ED, our findings may reflect a lack of uptake of the recommendations ED pharmacists made. While pharmacists routinely phoned and faxed written reports to community-based care providers, we were unable to collect information on process outcomes to understand whether communication between the medication review process and community-based prescribers was adequate to impact subsequent prescribing. Of four studies which previously measured outpatient health services utilization, the percentage of pharmacist recommendations that were adopted into practice ranged from 18 to 94% [25, 27, 28]. Future interventions should prioritize and evaluate the uptake of pharmacist recommendations by physicians to ensure that the proportion of recommendations accepted and adopted is high.

## Data Availability

The data were made available for the quality improvement evaluation of this study by the Vancouver Coastal Health Authority and the British Columbia Ministry of Health. Requests for data access can be sent to Joleen Wright, Director of Data Release Management, at Joleen.Wright@vch.ca.

## Abbreviations

ADE: Adverse Drug Events
BC: British Columbia
CTAS: Canadian Triage Acuity Score
ED: Emergency Department
GP: General Practitioner
LGH: Lions Gate Hospital
RH: Richmond Hospital
VGH: Vancouver General Hospital

## References

[1] Zed PJ, Abu-Laban RB, Balen RM (2008). Incidence, severity and preventability of medication-related visits to the emergency department: a prospective study. Can Med Assoc J.

[2] Budnitz DS, Lovegrove MC, Shehab N, Richards CL (2011). Emergency hospitalizations for adverse drug events in older Americans. N Engl J Med.

[3] Zed PJ, Abu-Laban RB, Balen RM, et al. Incidence, severity and preventability of medication-related visits to the emergency department: a prospective study. CMAJ. 2008;178(12):1563–9. 10.1503/cmaj.071594 PMC2396352 18519904

[4] Patel H, Bell D, Molokhia M (2007). Trends in hospital admissions for adverse drug reactions in England: analysis of national hospital episode statistics 1998-2005. BMC Clin Pharmacol.

[5] Hohl CM, Nosyk B, Kuramoto L (2011). Outcomes of Emergency Department Patients Presenting With Adverse Drug Events. Ann Emerg Med.

[6] Lazarou J, Pomeranz BH, Corey PN (1998). Incidence of adverse drug reactions in hospitalized patients: a meta-analysis of prospective studies. Jama.

[7] Hohl CM, Zed PJ, Brubacher JR, Abu-Laban RB, Loewen PS, Purssell R (2010). Do emergency physicians attribute drug-related emergency department visits to medication-related problems?. Ann Emerg Med.

[8] Hohl CM, Robitaille C, Lord V (2005). Emergency physician recognition of adverse drug-related events in elder patients presenting to an emergency department. Acad Emerg Med.

[9] Klopotowska JE, Wierenga PC, Smorenburg SM (2013). Recognition of adverse drug events in older hospitalized medical patients. Eur J Clin Pharmacol.

[10] Welk B, Richard L, Winick-Ng J, Shariff SZ, Clemens KK (2018). The Burden of Repeat Prescribing Medications after a Related Adverse Drug Event. Healthc Q.

[11] Clyne W, Blenkinsopp A, Seal R (2008). A guide to medication review.

[12] Accreditation Canada (2017). Required organizational practices handbook 2017.

[13] Christensen M, Lundh A. Medication review in hospitalised patients to reduce morbidity and mortality (review). Cochrane Libr. 2013;(2):CD008986. 10.1002/14651858.CD008986.pub2 23450593

[14] Hohl CM, Partovi N, Ghement I (2017). Impact of early in-hospital medication review by clinical pharmacists on health services utilization. PLoS One.

[15] Hohl CM, Brubacher J, Hunte G (2012). Clinical decision rules to improve the detection of adverse drug events in emergency department patients. Acad Emerg Med.

[16] Hohl CM, Badke K, Zhao A, et al. Prospective validation of clinical criteria to identify emergency department patients at high risk for adverse drug events. Acad Emerg Med. 2018. 10.1111/acem.13407. 10.1111/acem.13407 PMC6175415 29517818

[17] Hohl CM, McGrail K, Sobolev B (2015). The effect of pharmacist-led medication review in high-risk patients in the emergency department: an evaluation protocol. CMAJ Open.

[18] Hohl CM, Badke K, Zhao A (2018). Prospective validation of clinical criteria to identify emergency department patients at high risk for adverse drug events. Acad Emerg Med.

[19] Canadian Patient Safety Institute (2016). Medication Reconciliation.

[20] BC Vital Statistics Agency (2011). Vital Statistics Deaths. V2.

[21] BC Ministry of Health (2011). PharmaNet.

[22] British Columbia Ministry of Health. Medical Services Plan (MSP) Payment Information File. V2 2011; http://www.popdata.bc.ca/data. Accessed 20 Sept 2018.

[23] British Columbia Ministry of Health (2011). Discharge Abstract Database (Hospital Separations). V2.

[24] Wagner AK, Soumerai SB, Zhang F, Ross-Degnan D (2002). Segmented regression analysis of interrupted time series studies in medication use research. J Clin Pharm Ther.

[25] Lisby M, Bonnerup DK, Brock B (2018). Medication review and patient outcomes in an orthopedic department: a randomized controlled study. J Patient Saf.

[26] Okere AN, Renier CM, Tomsche JJ (2015). Evaluation of the influence of a pharmacist-led patient-centered medication therapy management and reconciliation service in collaboration with emergency department physicians. J Manag Care Spec Pharm.

[27] Gallagher P, O'Connor M, O'Mahony D (2011). Prevention of potentially inappropriate prescribing for elderly patients: a randomized controlled trial using STOPP/START criteria. Clinical Pharmacology & Therapeutics.

[28] Lisby M, Thomsen A, Nielsen LP (2010). The effect of systematic medication review in elderly patients admitted to an acute Ward of internal medicine. Basic Clin Pharmacol Toxicol.

[29] El Morabet N, Uitvlugt EB, van den Bemt BJF, van den Bemt PMLA, Janssen MJA, Karapinar-Çarkit F (2018). Prevalence and preventability of drug-related hospital readmissions: a systematic review. J Am Geriatr Soc.

[30] Garner R, Tanuseputro P, Manuel DG, Sanmartin C (2018). Transitions to long-term and residential care among older Canadians. Health Rep.

[31] Nickel CH, Ruedinger JM, Messmer AS (2013). Drug-related emergency department visits by elderly patients presenting with non-specific complaints. Scand J Trauma Resusc Emerg Med.

[32] Bernal JL, Cummins S, Gasparrini A (2016). Interrupted time series regression for the evaluation of public health interventions: a tutorial. Int J Epidemiol.

[33] Cortejoso L, Dietz R, Hofmann G, Gosch M, Sattler A (2016). Impact of pharmacist interventions in older patients: a prospective study in a tertiary hospital in Germany. Clin Interv Aging.

[34] Ziane A, Ngami C, Youb R (2013). Evaluating the quality of pharmacists’ interventions in older patient than 75 years. J Pharm Clin.

[35] Verdoorn S, Kwint H-F, Faber A, Gussekloo J, Bouvy ML (2015). Majority of drug-related problems identified during medication review are not associated with STOPP/START criteria. Eur J Clin Pharmacol.

[36] Somers A, Robays H, Vander Stichele R, Van Maele G, Bogaert M, Petrovic M (2010). Contribution of drug related problems to hospital admission in the elderly. J Nutr Health Aging.

[37] Onder G, Landi F, Liperoti R, Fialova D, Gambassi G, Bernabei R (2005). Impact of inappropriate drug use among hospitalized older adults. Eur J Clin Pharmacol.

[38] Budnitz DS, Shehab N, Kegler SR, Richards CL (2007). Medication use leading to emergency department visits for adverse drug events in older adults. Ann Intern Med.

[39] Santolaya-Perrín R, Calderón-Hernanz B, Jiménez-Díaz G, et al. The efficacy of a medication review programme conducted in an emergency department. Int J Clin Pharm. 2019;41(3):757–66. 10.1007/s11096-019-00836-0 31028596

[40] Beuscart J-B, Knol W, Cullinan S (2018). International core outcome set for clinical trials of medication review in multi-morbid older patients with polypharmacy. BMC Med.

[41] Hohl CM, Kuramoto L, Yu E, Rogula B, Stausberg J, Sobolev B (2013). Evaluating adverse drug event reporting in administrative data from emergency departments: a validation study. BMC Health Serv Res.

[42] Gillespie U, Alassaad A, Henrohn D (2009). A comprehensive pharmacist intervention to reduce morbidity in patients 80 years or older: a randomized controlled trial. Arch Intern Med.

[43] Lasota ST, Merrey W, Ross PA, Martin A, Feeser SA (2019). Provider responsiveness to pharmacist recommendations in a population health setting. Sr Care Pharm.

[44] Kripalani S, LeFevre F, Phillips CO, Williams MV, Basaviah P, Baker DW (2007). Deficits in communication and information transfer between hospital-based and primary care PhysiciansImplications for patient safety and continuity of care. JAMA.

